# Hepatobiliary manifestations in patients with ulcerative colitis: a retrospective analysis

**DOI:** 10.3389/fmed.2023.1273797

**Published:** 2024-01-05

**Authors:** Katharina Stratmann, Songül Aydolmus, Wenyi Gu, Dominik Heling, Ulrich Spengler, Birgit Terjung, Christian P. Strassburg, Richard Vollenberg, Irina Blumenstein, Jonel Trebicka

**Affiliations:** ^1^Goethe University Frankfurt, University Hospital, Medical Clinic 1, Frankfurt, Germany; ^2^Department of Internal Medicine I, University Hospital of Bonn, Bonn, Germany; ^3^Department of Internal Medicine B, University Clinic Münster, Münster, Germany

**Keywords:** inflammatory bowel disease, ulcerative colitis, hepatobiliary disorder, liver function test (LFT), risk factor

## Abstract

**Background:**

Inflammatory bowel diseases (IBDs) are often associated with altered liver function tests (LFTs). There is little data on the relationship between abnormal LFT and IBD. Our study aimed to evaluate the prevalence and etiology of elevated LFT in patients with ulcerative colitis (UC) and to determine whether there is an association with clinical and demographic parameters.

**Methods:**

The clinical records of the Gastroenterology Outpatients Clinic at a single center were reviewed and screened for patients with UC from 2005 to 2014. In total, 263 patients were included. Patients with Crohn’s disease (CD), colitis indeterminate, and colitis of other origins were excluded. Abnormal LFT and liver injuries were analyzed.

**Results:**

A cohort of 182 patients was analyzed (114 males, 68 females; mean age = 50.2 ± 16.1 years). 58 patients had already been diagnosed with a hepatobiliary disorder. Patients with a known hepatobiliary disorder suffered from UC for a significantly longer duration. Elevated LFT in patients without known hepatobiliary disorders was 69.4%. Liver injury was found in 21.8%. A transient increase in abnormal LFT was shown in 59 patients (68.6%), a persistent increase was found in 27 patients (31.4%). Treatment with thiopurines was a risk factor for persistent elevated LFT (*p* = 0.029), steroids had a protective impact (*p* = 0.037).

**Conclusion:**

This study clearly highlights the importance of screening for hepatobiliary disorders and abnormal LFT in patients with UC, as the prevalence of hepatobiliary disorders and abnormal LFT is detected very often among this patient group.

## Introduction

Extra-intestinal manifestations in patients with an inflammatory bowel disease (IBD), including hepatobiliary manifestations, are found in more than one-third of patients with IBD (1). Elevated liver function tests (LFTs) in IBD patients have been described in 11–49% of different studies (1–4). Chronic liver disease has even been reported in about 5% of adults with IBD (2). Primary sclerosing cholangitis (PSC) is the most common hepatobiliary manifestation, especially in patients with UC (5–7). Other hepatobiliary disorders, such as steatosis, autoimmune hepatitis (AIH), or primary biliary cholangitis (PBC), are less frequent (6, 7). In particular, the association between UC and PSC is relevant due to the increased risk of developing colorectal cancer and cholangiocarcinoma in very young patients (7–11).

The pathomechanisms behind the development of hepatobiliary disorders in patients with IBD are thought to be mainly immune mediated, but multifactorial genesis is probable. Aside from the altered immune response, drug side effects may also induce hepatobiliary diseases in patients with IBD (3, 12, 13). Besides corticosteroids and aminosalicylates, there are an increasing number of biologicals that can be used for the treatment of UC, for example, the monoclonal antibody Vedolizumab which is used to treat UC and other inflammatory conditions in the gastrointestinal tract (14). Biologicals and mesalazine in particular are potentially hepatotoxic, a consideration that should be taken into account before starting IBD treatment (7, 8). Especially, the tumor-necrosis factor-alpha inhibitor infliximab is well known for a drug-induced liver injury (DILI) (15). Due to these reasons and the suspected high rate of hepatobiliary disorders in patients with IBD, screening for these abnormalities is essential (2).

This study summarizes the most frequent hepatobiliary manifestations and the prevalence of LFT abnormalities in patients with UC followed up in our hands. Furthermore, we analyzed the risk factors or predictors of hepatobiliary disease or abnormalities in LFT in patients with UC.

## Materials and methods

This study was based on a retrospective screening of the clinical records of the Gastroenterology Outpatients Clinic at the Department of Internal Medicine I, University of Bonn. Patients with UC were screened in the time period from 2005 until 2014. The diagnosis of UC was determined according to standard clinical, radiological, endoscopic, and histological criteria.2.

In total, we included 263 patients. Patients with CD as well as indeterminate colitis and colitis of other origins were excluded. Additionally, we excluded patients who did not undergo follow-up. We considered a follow-up at three different points of times: (1) The patient appeared in the Gastroenterology Unit (outpatient and inpatient treatment) for the first time. (2) The second follow-up was during the monitoring period, when the liver enzymes were at a maximum. (3) The patient appeared in the Gastroenterology Unit for the last time in that monitoring period.

We used the Montreal Classification of the disease extent of UC to define disease features (1, 16).

An abnormality of LFT was defined as an increase in transaminases [alanine aminotransferase, ALT (=GPT); aspartate aminotransferase, AST (=GOT)], total bilirubin, gamma-glutamyl transpeptidase (GGT), or alkaline phosphatase (AP). We defined liver injury as an increase in ALT and AST of twice the upper limit normal (ULN).

Based on the relevant guidelines, the following methods for diagnosing NASH, alcohol-related liver disease, PBC, PSC, and AIH were used:

Nonalcoholic steatohepatitis was diagnosed if there was no anamnesis of alcohol abuse, a secondary steatosis was ruled out and if there was either an imaging or histological evidence of liver steatosis.

Alcohol related liver damage was diagnosed if there was an anamnesis of alcohol abuse and secondary steatosis was ruled out.

Primary biliary cholangitis was diagnosed if GGT and/or AP were repeatedly elevated, and anti-mitochondriale antibodies (AMA) or typical antinuclear antibodies (ANA) were positive.

Primary sclerosing cholangitis was diagnosed if GGT and/or AP were repeatedly elevated and after performing a MRCP or histological evidence of a PSC. Secondary causes of sclerosing cholangitis had to be excluded.

Autoimmune hepatitis was diagnosed if transaminases were repeatedly elevated, typical autoantibodies were positive and/or if there was histological evidence of an AIH. Viral hepatitis had to be ruled out.

### Statistical analysis

Continuous variables were described as means and standard deviations (SDs) and were compared using the Mann–Whitney U test. Categorical variables were shown as numbers with percentages and were compared using the *χ*^2^ test (chi-squared test). To compare potentially interacting covariates, a multivariate Cox regression model was used. All tests were two-tailed, and the level of significance was 5%. Statistical analysis was performed with SPSS (IBM SPSS Statistics, version 27).

### Ethical considerations

The ethical committee of the University of Bonn approved the study and the principle of a pseudonymized documentation (EK 254/14). All the principles outlined in the Declaration of Helsinki of 1975 have been followed in this retrospective study.

## Results

### General characteristics

Our study cohort included 263 patients. Patients with CD, indeterminate colitis, colitis of other origin, or no follow-up were excluded (81 patients; 30.8% of the entire cohort). A total of 182 patients with UC (69.2% of the entire cohort) were considered for analysis.

The study included 58 patients with UC and an already diagnosed hepatobiliary disorder and 124 patients without a known hepatobiliary disorder. A total of 114 (62.6%) male and 68 (37.4%) female patients were analyzed. The mean age of the cohort was 50.2 years, with a SD of 16.12 years (Table 1). The median duration of follow-up was 35 months.

**Table 1:** Demographic and clinical features of the analyzed cohort.

	Known liver disease	No liver disease	Total	*p* (Known liver disease vs. no liver disease)
Total	58 (%)	124 (%)	182 (%)	
Gender				
Male	39 (67.24)	75 (60.48)	114 (62.6)	0.380
Female	19 (32.76)	49 (39.52)	68 (37.4)	
Mean age	48.86 years (SD: 11.74)	50.9 years (SD: 17.81)	50.2 years (SD: 16.12)	0.361
Duration of disease				**< 0.001**
< 1 year	4 (6.9)	26 (20.97)	30 (16.5)	
1–5 years	6 (10.34)	27 (21.77)	33 (18.1)	
> 5 years	40 (68.97)	54 (43.55)	94 (51.6)	
unknown	8 (13.79)	17 (13.71)	25 (13.7)	
Montreal classification				0.764
E1	0 (0)	11 (8.87)	11 (6.0)	
E2	7 (12.07)	25 (20.16)	32 (17.6)	
E3	27 (46.55)	46 (37.1)	73 (40.1)	
Unknown	24 (41.38)	42 (33.87)	66 (36.3)	
Medical treatment				
Prednisolone	9 (15.52)	67 (54.03)	76 (41.8)	**0.004**
Azathioprine	3 (5.17)	28 (22.58)	31 (17.0)	**0.048**
Adalimumab	0 (0)	3 (2.42)	3 (1.6)	0.314
Infliximab	0 (0)	2 (1.61)	2 (1.1)	0.381
Budesonide	3 (5.17)	5 (4.03)	8 (4.4)	0.178
Mesalazine	14 (24.14)	82 (66.13)	96 (52.7)	**<0.001**
Abnormal LFT	57 (98.28)	86 (69.35)	143 (78.6)	**<0.001**
Liver injury (>2 x ULN of ALT and AST)	41 (70.69)	27 (21.77)	68 (37.4)	**<0.001**

Using Mann–Whitney-U- and chi-square-test for comparing known liver disease vs. no liver disease, *p* < 0.05. LFT, Liver function test; SD, Standard deviation; ULN, Upper limit normal. Bold values statistically significant with *p* < 0.05.

### Patients with abnormal LFT

In our cohort, the prevalence of elevated LFT in patients without known hepatobiliary disorders was 86 (69.4%). Liver injury, defined as an increase in LFT (ALT, AST) of more than 2-fold ULN (2XULN), was found in 27 (21.8%) patients.

The results showed a significant correlation between the presence of hepatobiliary disorder, abnormal LFT, and liver injury and the time since diagnosis of UC. Patients with known hepatobiliary disorders had a significantly higher rate of abnormal LFT and liver injury (*p* < 0.001) and a significantly longer duration of UC (*p* < 0.001). Importantly, there was no significant relationship between the presence of hepatobiliary disorders and the extent of UC at the initial diagnosis.

Furthermore, patients without known hepatobiliary disorders received significantly more often prednisolone, azathioprin, and mesalazine compared to patients with an already known hepatobiliary disorder (prednisolone: *p* = 0.004, 54.0 vs. 15.5%; azathioprine: *p* = 0.048, 22.6 vs. 5.2%; and mesalazine: *p* < 0.001, 66.1 vs. 24.1%; Table 1).

In patients with a diagnosed chronic liver disease, liver cirrhosis was already described in 55% of cases, of which 72% was due to PSC. The main hepatobiliary manifestation in our cohort was PSC (in total, in 79.3% of patients with UC and a chronic liver disease; Table 2). Table 2 shows the etiology of liver diseases in patients with a chronic liver disease.

**Table 2:** Summary of etiology of liver disease.

	*n* = 58 (%)	Hepatobiliary disorder *n* = 26 (%)	Liver cirrhosis *n* = 32 (%)
PSC	46 (79.3)	23 (88.5)	23 (71.9)
NASH	4 (6.9)	3 (11.5)	1 (3.1)
Viral hepatitis	3 (5.2) (2 hepatitis B, 1 hepatitis C)	0 (0)	3 (9.4) (2 hepatitis B, 1 hepatitis C)
AIH	3 (5.2)	0 (0)	3 (9.4)
Alcohol-related	2 (3.4)	0 (0)	2 (6.3)

AIH, Autoimmune hepatitis; NASH, Nonalcoholic steatohepatitis; PSC, Primary sclerosing cholangitis.

To further investigate the predictors and risk factors of increased LFT, we focused on patients without known hepatobiliary disorders (Table 3). Specifically, we compared patients with abnormal LFT, liver injury, and normal LFT. There was no significant difference associated with abnormal LFT, liver injury, normal LFT, and gender (*p* = 0.27), nor was there any significant difference in duration of disease (*p* = 0.31) or extent of disease (*p* = 0.96). In addition, we found no significant difference associated with increased LFT (abnormal LFT and liver injury) or medical treatment (Table 3).

**Table 3:** Analysis of risk factors and predictors for any abnormal liver function tests in patients without known liver diseases.

	Abnormal LFT (liver injury excluded) (*n* = 59) (%)	Liver injury (>2 x ULN of ALT and AST) (*n* = 27) (%)	Normal LFT (*n* = 38) (%)	*p* (abnormal LFT vs. liver injury vs. normal LFT)
Gender				0.269
Male	37 (62.71)	13 (48.15)	25 (65.79)	
Female	22 (37.29)	14 (51.85)	13 (34.21)	
Mean age	54.17 years (SD: 17.40)	49.04 years (SD: 17.44)	47.11 years (SD: 18.22)	0.199
Duration of disease				0.314
< 1 year	10 (16.95)	6 (22.22)	10 (26.32)	
1–5 years	15 (25.42)	2 (7.41)	10 (26.32)	
> 5 years	26 (44.07)	13 (48.15)	15 (39.47)	
Unknown	8 (13.56)	6 (22.22)	3 (7.89)	
Montreal classification				0.975
E1	5 (8.47)	2 (7.41)	4 (10.53)	
E2	10 (16.95)	5 (18.52)	10 (26.32)	
E3	21 (35.59)	9 (33.33)	16 (42.11)	
unknown	23 (38.98)	11 (40.74)	8 (21.05)	
Medical treatment				
Prednisolone	35 (59.32)	6 (22.22)	26 (68.42)	0.685
Azathioprine	17 (28.81)	3 (11.11)	8 (21.05)	0.163
Adalimumab	0 (0)	0 (0)	3 (7.89)	0.066
Infliximab	1 (1.69)	0 (0)	1 (2.63)	0.645
Budesonide	2 (3.39)	1 (3.70)	2 (5.26)	0.913
Mesalazine	43 (72.88)	10 (37.04)	29 (76.32)	0.943

Using the ANOVA test for comparing abnormal LFT vs. liver injury vs. normal LFT, *p* < 0.05. LFT, Liver function test; SD, Standard deviation; and ULN, Upper limit normal.

Furthermore, we differentiated between transient and persistent increase in LFT (Table 4). We defined a persistent increase as an increase with every follow-up appointment or an increase during the monitoring period plus an increase at the end, at least for one LFT. The rest was defined as a transient increase. A transient increase in abnormal LFT was found in 59 patients (68.6%). A persistent increase was observed in 27 patients (31.4%). Gender, age, duration, and extent of disease did not show any significance. However, taking prednisolone had a protective impact (*p* = 0.037), while azathioprine was a risk factor for persistent elevated LFT (*p* = 0.029; Table 4).

**Table 4:** Analysis of risk factors and predictors for any abnormal LFT with a transient or persistent increase.

	Transient increase (*n* = 59) (%)	Persistent increase (*n* = 27) (%)	Normal LFT (*n* = 38) (%)	*p* (persistent increase as outcome)	Regression coefficient B	SD	OR	95% CI
Gender								
Male	33 (55.9)	17 (63)	25 (65.8)	0.2	−0.407	0.318	0.665	0.357, 1.241
Female	26 (44.1)	10 (37)	13 (34.2)					
Mean age	51.14 years (SD: 17.53)	55.7 years (SD: 17.25)	47.11 years (SD: 18.22)	0.46	0.006	0.009	1.006	0.989, 1.024
Duration of disease								
< 1 year	11 (18.6)	5 (18.5)	10 (26.3)	0.46	−0.158	0.215	0.854	0.561, 1.301
1–5 years	14 (23.7)	3 (11.1)	10 (26.3)					
> 5 years	28 (47.5)	11 (40.7)	15 (39.5)					
Unknown	6 (10.2)	8 (29.6)	3 (7.9)					
Montreal classification								
E1	4 (6.8)	3 (11.1)	4 (10.5)	0.7	0.107	0.279	1.113	0.645, 1.921
E2	12 (20.3)	3 (11.1)	10 (26.3)					
E3	20 (33.9)	10 (37.0)	16 (42.1)					
Unknown	23 (39.0)	11 (40.7)	8 (21.1)					
Medical treatment								
Prednisolone	30 (50.8)	11 (40.7)	26 (68.4)	**0.037**	−0.78	0.374	0.459	0.220, 0.954
Azathioprine	18 (30.5)	2 (7.4)	8 (21.1)	**0.029**	0.763	0.349	2.145	1.083, 4.247
Adalimumab	0 (0)	0 (0)	3 (7.9)	0.98	−14.7	609.92	0	0
Infliximab	0 (0)	1 (3.7)	1 (2.6)	0.99	−13.11	772.54	0	0
Budesonide	2 (3.4)	1 (3.7)	2 (5.3)	0.11	−1.273	0.786	0.280	0.060, 1.305
Mesalazine	39 (66.1)	14 (51.9)	29 (76.3)	0.25	−0.465	0.407	0.628	0.283, 1.396

Using multivariable Cox regression with persistent increase as outcome, *p* < 0.05. CI, Confidence interval; LFT, Liver function test; OR, Odds ratio; and SD, Standard deviation. Bold values statistically significant with *p* < 0.05.

Additionally, we analyzed clinical symptoms, such as the frequency of diarrhea and abdominal pain that may have an influence on persistent elevated LFT. These symptoms are often described by patients with an acute flare-up of UC. In our cohort, there was no significant difference between clinical symptoms and the persistent increase in LFT (Table 5). Here, we also conducted a multivariate analysis using the Cox regression with persistent liver dysfunction as the outcome (Table 5).

**Table 5:** Analysis for clinical symptoms as risk factors for any abnormal liver function tests.

	Regression coefficient *B*	SD	*p*	OR	95% CI
Frequency of diarrhea	0.024	0.025	0.335	1.024	0.975, 1.076
Abdominal pain	0.365	0.4	0.361	1.441	0.658, 3.158

Using multivariate Cox regression with persistent increase as outcome, *p* < 0.05. CI, Confidence interval; OR, Odds ratio; and SD, Standard deviation.

### Screening algorithm

**Figure 1:**
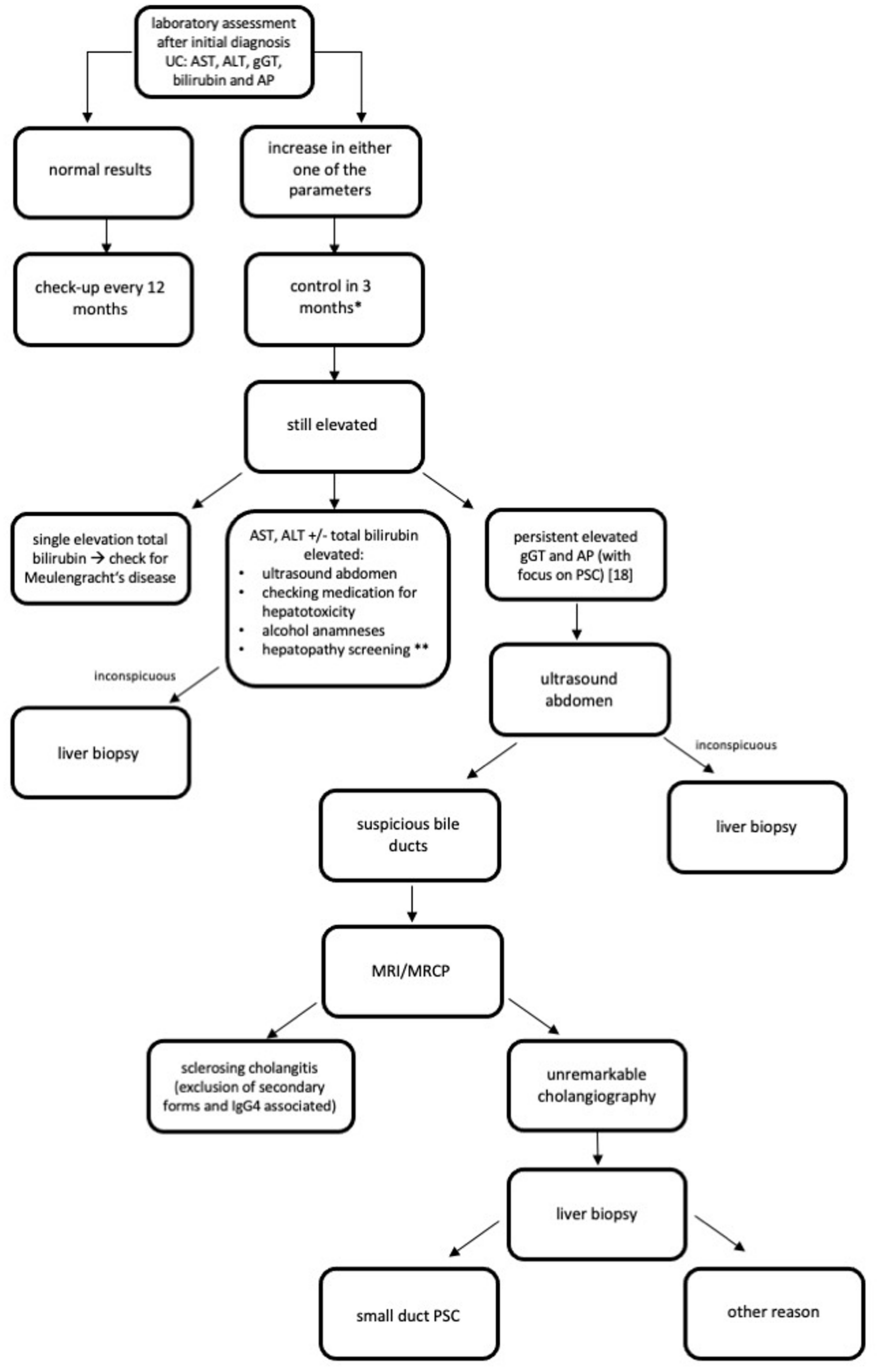
Recommended algorithm for screening of liver diseases after initial diagnosis CU (33). AIH, Autoimmune hepatitis; ALT, Alanine aminotransferase; AP, Alkaline phosphatase; AST, Aspartate aminotransferase; gGT, Gamma-glutamyl transpeptidase; HEV, Hepatitis E; HIV, Human immunodeficiency virus; HSV, Herpes simplex virus; MRCP, Magnetic resonance cholangiopancreatography; MRI, Magnet resonance imaging; NASH, Nonalcoholic steatohepatitis; PBC, Primary biliary cholangitis; PSC, Primary sclerosing cholangitis; UC, Ulcerative Colitis; ULN, Upper limit normal. ^*^if liver synthesis parameter are affected and transaminases > 2xULN or clinical symptoms as jaundice and confusion appear, please check earlier and think of hospitalization. ^**^Hepatopathy screening: viral hepatitis: hepatitis A, B, and C (perhaps investigate for further infective causes, e.g., HEV, HIV, and HSV); AIH, PSC, and PBC; Hereditary hepatitis as Wilson’s disease, alpha-1-antitrypsin-deficiency, and hemochromatosis; and NASH.

Since there is a high rate of abnormal LFT and hepatobiliary disorders in patients with UC, screening for a liver dysfunction is essential and should be conducted regularly. We recommend using a special screening algorithm for every patient diagnosed with UC. We designed an algorithm for patients being treated in an outpatient clinic (Figure 1). Mazza et al. (17) designed an algorithm for patients with an IBD having abnormal LFT. We recommend screening for abnormal LFT in every patient with UC every 12 months when the initial assessment is inconspicuous. If there is abnormal LFT, hepatocellular and cholestasis patterns should be differentiated. Triggers, especially hepatotoxicity caused by UC medication, should be evaluated. An imaging procedure is also important. When laboratory assessment, anamneses, and imaging procedures do not offer a clear picture of the disease, we recommend a liver biopsy. A summary of this stepwise approach is shown in Figure 1.

## Discussion

Several studies have described elevated LFT in patients with an IBD (2, 18, 19). The prevalence of elevated LFT can be different in these studies depending on the criteria used to define abnormal LFT.

In our cohort, 98.3% with UC and an already diagnosed hepatobiliary disorder and 70.7% with a liver injury had abnormal LFT. Abnormal LFT is usually caused by liver dysfunction. However, increased LFT was found in 69.4% with no existing hepatobiliary disorder and 21.8% with liver injuries (Table 1). This underlines the importance of screening for elevated LFT regularly in patients with UC, even if there is no known proven predisposition for a liver dysfunction. This supports the hypothesis that UC and the resulting inflammation could be factors in the onset of liver damage.

Patients with a known hepatobiliary disorder had suffered UC for significantly longer than patients without (*p* < 0.001; Table 1). A multicenter study by Riegler et al. (19) showed that diffuse liver damage without a history of liver disease in patients with IBD (also CD and UC) is not rare. Damage was shown to correlate with the duration of the disease, to the activity index of the disease, and treatment with steroids and mesalazine (19). Therefore, we assume the longer patients suffer from UC, the higher the risk for liver damage. Multiple reasons for this hypothesis may be considered. For example, medical treatment, altered immune responses in patients with UC, duration of inflammation, and age at initial diagnosis are all factors that could increase the risk of liver damage. But further prospective studies are needed for confirmation.

In contrast to study of Riegler et al. (19), our analyses did not show any correlations between abnormal LFT or liver injury in patients without a hepatobiliary disorder and the duration of the disease or special medical treatments (Table 3). We explain this difference by highlighting that there was missing information in our data set and that Riegler et al. (19) used different investigation methods in their analysis. Due to missing data, we considered only laboratory parameters for our analysis. However, given the prevalence of elevated LFT in our cohort, we still consider regular testing to be essential. However, further prospective studies are needed to confirm this recommendation.

The most common hepatobiliary manifestation is PSC. Steatosis, AIH, or PBC are less common (6, 7). In our study, 58 patients already had an existing hepatobiliary disorder. In addition, PSC was the most frequent hepatobiliary disorder in our cohort as well. In 23 of the 46 patients with PSC, PSC-associated liver cirrhosis had already been diagnosed. Nonalcoholic steatohepatitis was found in a low proportion. And a viral hepatitis was found in a neglectable number (Table 2). Among patients with UC, about 4–7% develop PSC (20, 21). The difference between prior results and our data might be explained by the specialization of Bonn’s Gastroenterology Unit, which focuses on hepatologic diseases and not on patients with an IBD.

In our study, the prevalence of elevated LFT in patients without a known hepatobiliary disorder was higher than in other studies (69.4 vs. 11–49%; Table 1) (2, 18, 19). An analysis by Schrumpf et al. (22) described hepatobiliary complications in 3–15%. One reason for the different results might be, again, the specialization of Bonn’s Gastroenterology Unit. Other reasons may be the difference in the definition of abnormal LFT among different studies, as well as the different diagnostic methods used. We defined abnormal LFT as an increase in transaminases, total bilirubin, GGT, or AP. In 69.4% of patients, elevated LFT was measured at least once at some time during the follow-up period.

In our cohort, transient elevations in LFT (abnormal LFT and liver injury) were detected in 68.6%. Persistent elevations were found in 31.4% of the patients (Table 4). A study by Cappello et al. (3) showed transient elevations in LFT returning to normal levels within 3 months in 62.9% of patients. Persistent elevations were detected in 37.1% (3). The results of a large Swedish follow-up study were similar to the results of the study by Cappello et al. (23). In our study, the average time period of the follow-up was 35 months. Perhaps due to the longer follow-up period, more cases of abnormal LFT returning to normal could be found. The cause of transient abnormal LFT could not be evaluated in our study due to missing data.

In our study, azathioprine was a risk factor for persistent elevated LFT, while prednisolone had a protective impact on LFT (Table 4). Thiopurines are known to contribute to liver toxicity in a dose-dependent or dose-independent manner. Many IBD therapies, e.g., infliximab may cause liver toxicity, so DILI is a relevant issue for patients with IBD. Thiopurines, methotrexate, and infliximab are mostly associated with DILI (15, 17, 24, 25). Steroids (in our study, prednisolone) had a protective impact. Steroids are used in IBD treatment and the management of hepatobiliary disorders in IBD (e.g., in patients with AIH or IgG4-related cholangitis) (26). The protective impact of prednisolone could be explained by a suppression of immune activation contributing to further immune-mediated complications. However, as steroids could also cause hepatic complications, such as the reactivation of HBV and steatohepatitis (26), a careful use is still essential. Additionally, the protective effect of prednisone might reflect a not yet diagnosed autoimmune hepatitis partially treated. For future investigation, an AIH score could consider the response to therapy.

These results again underline the importance of regularly monitoring liver function, especially when patients are undergoing certain medical treatments.

Additionally, we assessed clinical symptoms, such as the frequency of diarrhea and abdominal pain. In our cohort, we could not find a correlation between these clinical symptoms and a persistent increase in LFT (Table 5). This could lead to the assumption that clinical parameters do not have an influence on LFT. Due to the subjectivity of these parameters, we assume that they could cause a mismatch and do not reflect the activity index in UC patients. Endoscopic scores, such as the Mayo Clinic Endoscopic Subscore (MAYO Score) and the UC Endoscopic Index of Severity (UCEIS) score, or the fecal calprotectin (fCP) are valuable parameters for determining the activity index in patients with UC (27–32). Due to missing data in our cohort, we could not take these into account.

The most important limitation of our study is its retrospective design. Due to the specialization of Bonn’s Gastroenterology Unit, a selection bias may be another important limitation as hepatobiliary disorders may be overrepresented. Moreover, there were some missing data that could have affected the analysis.

Since in our study, LFT was frequently found in patients with IBD without clear causality, this study clearly highlights the importance of screening for hepatobiliary disorders and abnormal LFT in patients with IBD, as well as careful selection of treatment. Especially laboratory assessments that should be conducted regularly for each patient. We recommend laboratory assessments for ALT, AST, GGT, AP, and bilirubin every 3–6 months. Additionally, further examinations, such as ultrasound of the abdomen and magnetic resonance imaging (MRI), are important for the screening of hepatologic changes. But in our opinion, our study serves as an upfront reference and basis for further prospective investigations.

We found a correlation between the duration of UC and the occurrence of a hepatobiliary disease. Patients with a longer duration of UC had a hepatobiliary disorder significantly more often. Therefore, we recommend that patients with a longer duration of UC be screened for hepatologic changes more often.

We hypothesize that screening for hepatobiliary disorders is necessary for patients with UC; therefore, we recommend using the special screening algorithm designed by us after the initial diagnosis of UC (Figure 1).

Since hepatobiliary disorders are often found in patients with UC, prospective studies evaluating the risk factors and predictors of hepatobiliary disorders are needed for patients with IBD. Finding potential risk factors and predictors could enhance preventive measures and enhance care for patients with IBD.

## Data availability statement

The raw data supporting the conclusions of this article will be made available by the authors, without undue reservation.

## Ethics statement

The studies involving humans were approved by the ethical committee of the University of Bonn approved the study and the principle of a pseudonymized documentation (EK 254/14). All the principles outlined in the Declaration of Helsinki of 1975 have been followed in this retrospective study. The studies were conducted in accordance with the local legislation and institutional requirements. The human samples used in this study were acquired from a by-product of routine care or industry. Written informed consent for participation was not required from the participants or the participants’ legal guardians/next of kin in accordance with the national legislation and institutional requirements.

## Author contributions

KS: Conzeptualization, Data curation, Formal analysis, Visualization, Writing - original draft. SA: Data curation, Writing – original draft. WG: Formal Analysis, Writing – review & editing. DH: Writing – review & editing. US: Writing – review & editing. BT: Writing – review & editing. CS: Writing – review & editing. RV: Writing – review & editing. IB: Conceptualization, Writing – review & editing. JT: Conceptualization, Project administration, Supervision, Writing – review & editing.

## Figures and Tables

**Table:** 

AIH	Autoimmune hepatitis
ALT (=GPT)	Alanine aminotransferase
AMA	Anti-mitochondriale antibody
ANA	Antinuclear antibody
AP	Alkaline phosphatase
AST (=GOT)	Aspartate aminotransferase
CD	Crohn’s disease
CI	Confidence interval
DILI	Drug-induced liver injury
e.g.	Exempli gratia
fCP	Fecal calprotectin
GGT	Gamma-glutamyl transpeptidase
HEV	Hepatitis E
HIV	Human immunodeficiency virus
HSV	Herpes simplex virus
IBD	Inflammatory bowel disease
LFT	Liver function test
MAYO	Mayo clinic endoscopic subscore
MRCP	*Magnetic resonance cholangiopancreatography*
MRI	Magnet resonance imaging
NASH	Nonalcoholic steatohepatitis
OR	Odds ratio
PBC	Primary biliary cholangitis
PSC	Primary sclerosing cholangitis
SD	Standard deviation
UC	Ulcerative colitis
UCEIS	Ulcerative Colitis Endoscopic Index of Severity
ULN	Upper limit normal

## References

[1] Ott C Scholmerich J. Extraintestinal manifestations and complications in IBD. Nat Rev Gastroenterol Hepatol. (2013) 10:585–95. doi: 10.1038/nrgastro.2013.117 23835489

[2] Mendes FD Levy C Enders FB Loftus EV Angulo P Lindor KD. Abnormal hepatic biochemistries in patients with inflammatory bowel disease. Am J Gastroenterol. (2007) 102:344–50. doi: 10.1111/j.1572-0241.2006.00947.x 17100965

[3] Cappello M Randazzo C Bravatà I Licata A Peralta S Craxì A. Liver function test abnormalities in patients with inflammatory bowel diseases: a hospital-based survey. Clin Med Insights Gastroenterol. (2014) 7:CGast.S13125. doi: 10.4137/CGast.S13125 PMC4069044 24966712

[4] Núñez FP Quera R Bay C Castro F Mezzano G. Drug-induced liver injury used in the treatment of inflammatory bowel disease. J Crohns Colitis. (2022) 16:1168–76. doi: 10.1093/ecco-jcc/jjac013 35044449

[5] Smith MP Loe RH. Sclerosing cholangitis. Review of recent case reports and associated diseases and four new cases. Am J Surg. (1965) 110:519–26. doi: 10.1016/0002-9610(65)90018-8, PMID: 14313190

[6] Yarur AJ Czul F Levy C. Hepatobiliary manifestations of inflammatory bowel disease. Inflamm Bowel Dis. (2014) 20:1655–67. doi: 10.1097/MIB.0000000000000065 24874461

[7] Fousekis FS Theopistos VI Katsanos KH Tsianos EV Christodoulou DK. Hepatobiliary manifestations and complications in inflammatory bowel disease: a review. Gastroenterol Res. (2018) 11:83–94. doi: 10.14740/gr990w, PMID: 29707074 PMC5916631

[8] Losurdo G Brescia IV Lillo C Mezzapesa M Barone M Principi M. Liver involvement in inflammatory bowel disease: what should the clinician know? World J Hepatol. (2021) 13:1534–51. doi: 10.4254/wjh.v13.i11.1534, PMID: 34904028 PMC8637677

[9] Zheng HH Jiang XL. Increased risk of colorectal neoplasia in patients with primary sclerosing cholangitis and inflammatory bowel disease: a meta-analysis of 16 observational studies. Eur J Gastroenterol Hepatol. (2016) 28:383–90. doi: 10.1097/MEG.0000000000000576 26938805

[10] Ngu JH Gearry RB Wright AJ Stedman CAM. Inflammatory bowel disease is associated with poor outcomes of patients with primary sclerosing cholangitis. Clin Gastroenterol Hepatol. (2011) 9:1092–7. doi: 10.1016/j.cgh.2011.08.027 21893134

[11] Navaneethan U Venkatesh PGK Lashner BA Shen B Kiran RP. The impact of ulcerative colitis on the long-term outcome of patients with primary sclerosing cholangitis. Aliment Pharmacol Ther. (2012) 35:1045–53. doi: 10.1111/j.1365-2036.2012.05063.x 22428605

[12] Navaneethan U Shen B. Hepatopancreatobiliary manifestations and complications associated with inflammatory bowel disease. Inflamm Bowel Dis. (2010) 16:1598–619. doi: 10.1002/ibd.21219, PMID: 20198712

[13] Björnsson ES Gu J Kleiner DE Chalasani N Hayashi PH Hoofnagle JH. Azathioprine and 6-Mercaptopurine-induced liver injury. J Clin Gastroenterol. (2017) 51:63–9. doi: 10.1097/MCG.0000000000000568, PMID: 27648552 PMC5505863

[14] Li ACW Dong C Tay ST Ananthakrishnan A Ma KSK. Vedolizumab for acute gastrointestinal graft-versus-host disease: a systematic review and meta-analysis. Front Immunol. (2022) 13:1025350. doi: 10.3389/fimmu.2022.1025350 36439135 PMC9692080

[15] Björnsson HK Gudbjornsson B Björnsson ES. Infliximab-induced liver injury: clinical phenotypes, autoimmunity and the role of corticosteroid treatment. J Hepatol. (2022) 76:86–92. doi: 10.1016/j.jhep.2021.08.024 34487751

[16] Satsangi J Silverberg MS Vermeire S Colombel JF. The Montreal classification of inflammatory bowel disease: controversies, consensus, and implications. Gut. (2006) 55:749–53. doi: 10.1136/gut.2005.082909, PMID: 16698746 PMC1856208

[17] Mazza S Soro S Verga MC Elvo B Ferretti F Cereatti F. Liver-side of inflammatory bowel diseases: hepatobiliary and drug-induced disorders. World J Hepatol. (2021) 13:1828–49. doi: 10.4254/wjh.v13.i12.1828 35069993 PMC8727201

[18] Heikius B Niemelä S Lehtola J Karttunen T Lähde S. Hepatobiliary and coexisting pancreatic duct abnormalities in patients with inflammatory bowel disease. Scand J Gastroenterol. (1997) 32:153–61. doi: 10.3109/00365529709000186, PMID: 9051876

[19] Riegler G D’Incà R Sturniolo GC Corrao G Del Vecchio BC Di Leo V. Hepatobiliary alterations in patients with inflammatory bowel disease: a multicenter study. Scand J Gastroenterol. (1998) 33:93–8. PMID: 9489915 10.1080/00365529850166275

[20] Rojas-Feria M Castro M Suárez E Ampuero J Romero-Gómez M. Hepatobiliary manifestations in inflammatory bowel disease: the gut, the drugs and the liver. World J Gastroenterol. (2013) 19:7327–40. doi: 10.3748/wjg.v19.i42.7327, PMID: 24259964 PMC3831215

[21] Broomé U Bergquist A. Primary sclerosing cholangitis, inflammatory bowel disease, and colon cancer. Semin Liver Dis. (2006) 26:031–41. doi: 10.1055/s-2006-933561 16496231

[22] Schrumpf E Fausa O Elgjo K Kolmannskog F. Hepatobiliary complications of inflammatory bowel disease. Semin Liver Dis. (1988) 8:201–9. doi: 10.1055/s-2008-1040541 3068805

[23] Broomé U Glaumann H Hellers G Nilsson B Sörstad J Hultcrantz R. Liver disease in ulcerative colitis: an epidemiological and follow up study in the county of Stockholm. Gut. (1994) 35:84–9. doi: 10.1136/gut.35.1.84, PMID: 8307457 PMC1374638

[24] Tran-Minh ML Sousa P Maillet M Allez M Gornet JM. Hepatic complications induced by immunosuppressants and biologics in inflammatory bowel disease. World J Hepatol. (2017) 9:613. doi: 10.4254/wjh.v9.i13.613 28539989 PMC5424291

[25] Bastida G Nos P Aguas M Beltrán B Rubín Á Dasí F. Incidence, risk factors and clinical course of thiopurine-induced liver injury in patients with inflammatory bowel disease. Aliment Pharmacol Ther. (2005) 22:775–82. doi: 10.1111/j.1365-2036.2005.02636.x 16225485

[26] Yaccob A Mari A. Practical clinical approach to the evaluation of hepatobiliary disorders in inflammatory bowel disease. Frontline Gastroenterol. (2019) 10:309–15. doi: 10.1136/flgastro-2018-101037, PMID: 31281626 PMC6583566

[27] Travis SPL Schnell D Krzeski P Abreu MT Altman DG Colombel JF. Developing an instrument to assess the endoscopic severity of ulcerative colitis: the ulcerative colitis endoscopic index of severity (UCEIS). Gut. (2012) 61:535–42. doi: 10.1136/gutjnl-2011-300486, PMID: 21997563 PMC3292713

[28] Schroeder KW Tremaine WJ Ilstrup DM. Coated Oral 5-Aminosalicylic acid therapy for mildly to moderately active ulcerative colitis. N Engl J Med. (1987) 317:1625–9. doi: 10.1056/NEJM198712243172603 3317057

[29] Roseth AG Aadland E Grzyb K. Normalization of faecal calprotectin: a predictor of mucosal healing in patients with inflammatory bowel disease. Scand J Gastroenterol. (2004) 39:1017–20. doi: 10.1080/00365520410007971, PMID: 15513345

[30] Schoepfer AM Beglinger C Straumann A Trummler M Renzulli P Seibold F. Ulcerative colitis:correlation of the Rachmilewitz endoscopic activity index with fecal calpro- tectin, clinical activity, C-reactive protein, and blood leukocytes. Inflamm Bowel Dis. (2009) 15:1851–8. doi: 10.1002/ibd.20986 19462421

[31] Mao R Xiao Y Gao X Chen BL He Y Yang L. Fecal calprotectin in predicting relapse of inflammatory bowel diseases: a meta-analysis of prospective studies. Inflamm Bowel Dis. (2012) 18:1894–9. doi: 10.1002/ibd.22861, PMID: 22238138

[32] Lin JF Chen JM Zuo JH Yu A Xiao ZJ Deng FH. Meta-analysis: fecal calprotectin for assessment of inflammatory bowel disease activity. Inflamm Bowel Dis. (2014) 20:1407–15. doi: 10.1097/MIB.0000000000000057 24983982

[33] Schramm CS (2017). Practice guideline autoimmune liver diseases. AWMF. Available at: https://www.awmf.org/uploads/tx_szleitlinien/021-027l_S2k_Autoimmune_Lebererkrankungen_2017-11.pdf 10.1055/s-0043-120199 29141269

